# Structure-Based Function of Humic Acid in Abiotic Stress Alleviation in Plants: A Review

**DOI:** 10.3390/plants14131916

**Published:** 2025-06-22

**Authors:** Farhan Nabi, Ahmed Sarfaraz, Rakhwe Kama, Razia Kanwal, Huashou Li

**Affiliations:** 1 College of Natural Resources and Environment, South China Agricultural University, Guangzhou 510643, China; khosofarhan23@gmail.com (F.N.); krakhwe@yahoo.fr (R.K.); 2 School of Life Sciences and Engineering, Southwest University of Science and Technology, Mianyang 621010, China; sarfarazahmed7977@gmail.com; 3 Department of Biotechnology, Sardar Bahadur Khan Women University, Quetta 87300, Pakistan; raziakanwal55@yahoo.com

**Keywords:** antioxidant activity, humic acid, soil fertility

## Abstract

Humic acid (HA), a major component of soil organic matter, is a naturally occurring macromolecule formed through the decomposition of plant and microbial residues. Its molecular structure comprises functional groups such as carboxyl, phenolic, hydroxyl, and carbonyl functional groups, which enable HA to interact with soil particles, nutrients, and biological systems. These interactions significantly contribute to soil fertility and overall plant productivity. Functionally, HA enhances soil health by increasing cation exchange capacity, improving water retention, and promoting the formation and stabilization of soil aggregates. In addition to its role in soil conditioning, HA is essential in mitigating plant stress. It achieves this by modulating antioxidant enzyme activity, stabilizing cellular membranes, and alleviating the adverse effects of abiotic stressors such as salinity, drought, and heavy metal toxicity. This review highlights the structural characteristics of HA, its structure-based functions, and the mechanisms involved in plant stress alleviation. Additionally, we explore how HA can be modified through physical, chemical, and biological approaches to enhance its agronomic performance. These modifications are designed to improve HA agronomic efficiency by increasing nutrient bioavailability, reducing environmental losses through minimized leaching and volatilization, and supporting sustainable agricultural practices. Overall, this review underscores the multifaceted roles of HA in promoting plant resilience to environmental stress, highlighting its potential as a key agent in the development of sustainable and eco-friendly crop production systems.

## 1. Introduction

Humic substances (HS) are complex organic molecules produced by the natural breakdown and subsequent microbial and abiotic transformation of plant and animal materials, including components such as cellulose, lignin, cutins, tannins, and chitins [1,2,3]. HS are vital for soil health because they improve nutrient retention, soil structure, and microbial activity. HS typically accumulate in soil through the decomposition and transformation of organic materials such as crop residues, forest litter, and other plant or microbial matter that is either deliberately returned to the soil or naturally incorporated through decomposition processes [4]. However, the growing demand for crop residues in alternative applications, such as animal feed, bioenergy, and biogas production has significantly disrupted this natural cycle. Instead of being returned to the soil, these residues are increasingly diverted, resulting in a substantial decline in the organic matter available for the formation of HS [5,6,7]. As a result, HS levels in agricultural soils are declining, posing a significant threat to key soil functions such as fertility, water retention, and carbon sequestration. While traditionally regarded as mere end products of organic matter decomposition, emerging research suggests that HS may also be synthesized through biochemical pathways including polymerization, polycondensation, and molecular rearrangement. This ongoing discourse within the scientific community highlights that HS possess both degradative and synthetic origins, contributing to their structural complexity and functional diversity [3,8]. This trend highlights the urgent need for strategies to balance resource use and restore organic matter to the soil for plant growth and productivity.

**Figure 1:**
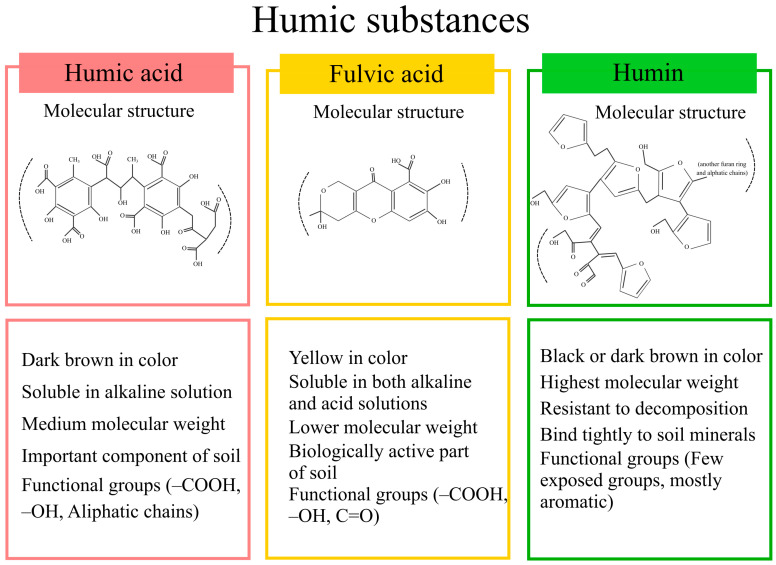
General representation of humic substances, including their molecular structure, functional groups, and associated biological functions. The molecular models of humic acid, fulvic acid, and humin are adapted from PubChem [27].

Humic substances are primarily composed of three fractions: humins, HA, and fulvic acid (FA). Humins are highly heterogeneous and recalcitrant fractions of soil organic matter that remain insoluble in both organic and inorganic solvents across the entire pH spectrum [9]. They consist of various substructures, including condensed aromatic and aliphatic components (such as alkyl and carbohydrate-derived compounds), and are often strongly bound to soil minerals. Their degradation rates vary depending on environmental conditions [10]. Due to their insolubility and limited biological reactivity, humins receive less attention compared to FA and HA, which are more readily available to interact with soil systems and thus hold greater potential for enhancing soil fertility. FA and HA exhibit both chemical reactivity and resistance to microbial degradation, making them highly beneficial to soil and plant systems (Figure 1) [11]. HA, in particular, demonstrates long-term stability against decomposition and possesses amphiphilic properties, enabling the formation of organo-mineral complexes. HA typically contains around 60% organic carbon, along with essential elements such as oxygen, hydrogen, nitrogen, and sulfur [12]. For instance, Gerke [8] demonstrated that HS complexes facilitate the mobilization of phosphorus (P) from iron- and aluminum-bound forms, while Urrutia et al. [13] highlighted the role of HS in enhancing plant acquisition of iron and sulfur. These interactions significantly improve nutrient bioavailability and uptake efficiency, contributing to more sustainable crop productivity. Moreover, HA improves soil structure and water retention by enhancing aggregate stability [14,15,16]. It also increases the availability of nutrients, particularly micronutrients, through complexation with carboxyl and phenolic functional groups. This leads to the formation of humates and stable salts that help regulate the release and uptake of nutrients over time [17]. This process reduces nutrient immobilization in the soil, thereby increasing the bioavailability of micronutrients to plants [18]. Additionally, it facilitates the precipitation of toxic heavy metals, limiting their mobility and reducing their concentrations in the soil. HA also enhances the activity of plant growth-promoting hormones such as auxins and cytokinins, which play critical roles in regulating photosynthesis, nutrient metabolism, and stress tolerance [11,19,20,21]. However, some studies have reported limited or inconsistent effects of HA application on crop growth and soil physicochemical properties [22,23,24]. While higher concentrations of HA have been associated with improvements in soil physical properties such as aggregate stability and porosity [25], the overall impact on soil chemical properties and crop performance remains inconclusive and warrants further investigation [26].

Humic acid promotes plant growth through a combination of physiological and biochemical mechanisms. It alters plasma membrane transporters, enhancing ion exchange and facilitating improved nutrient absorption, assimilation, and overall metabolism. By stimulating root cell elongation and division, HA promotes the development of an extensive root system, which increases the plant’s capacity for water and nutrient uptake. Its low-molecular-weight fractions exhibit hormone-like characteristics, mimicking the activity of natural plant hormones such as auxins and cytokinins. These compounds influence molecular signaling pathways that regulate key growth processes, including cell division, shoot development, and photosynthesis [28]. HA possesses various functional groups that contribute to plant defense mechanisms. The antimicrobial properties observed in HS, in part, are influenced by their oxygen-containing functional groups, such as carboxyl and phenolic groups, which contribute to overall chemical reactivity. These characteristics can help suppress soil-borne pathogens and support beneficial microbial communities, thereby reducing the incidence and severity of certain plant diseases. Phenolic groups in HA play a key role in neutralizing free radicals, forming a protective barrier that shields plant cells from oxidative damage caused by reactive oxygen species (ROS) [18]. Compounds containing carboxyl groups, particularly under alkaline conditions, may exhibit antioxidant and anti-inflammatory properties that help plants mitigate stress responses [29]. Quinone groups, another important component of HA, contribute to ROS scavenging and further reduce oxidative stress [30]. Additionally, the aromatic domains within HA (whose atomic O/C ratios can range from near zero in highly diagenetically altered forms to around 0.45 in lignin-derived fractions), with likewise variable H/C ratios, are associated with enhanced plant tolerance to various diseases [31,32]. Chemical compounds containing functional groups such as carboxyl (C=O and –OH), hydroxyl (–OH), and quinone (C=O) may exhibit antimicrobial properties, depending on their overall molecular context. Overall, the biological activity of HAs are strongly linked to their molecular structure, chemical composition, and physical properties, all of which collectively contribute to their physiological effects on plants [30,33,34,35,36,37,38].

**Figure 2:**
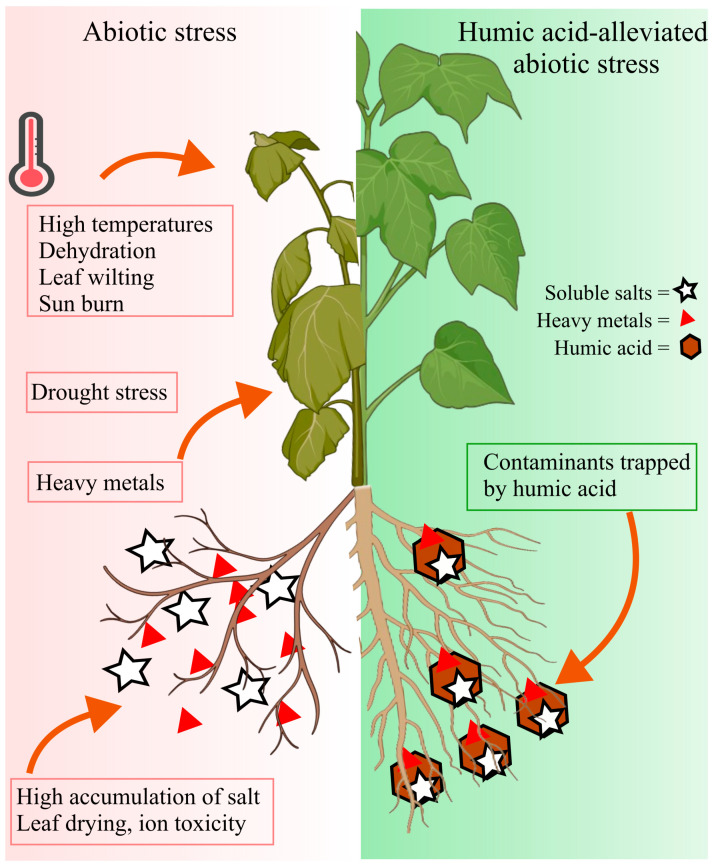
Effects of various abiotic stresses (salinity, drought, extreme temperature, and heavy metals) on morphological, physiological, and biochemical attributes of plants.

Plants are vulnerable to a wide range of environmental stressors, including salinity, drought, extreme temperatures, and toxic metals (Figure 2) [5]. HA has emerged as a promising biostimulant for mitigating the effects of these abiotic stresses while simultaneously enhancing crop quality and yield. Its complex molecular structure and multifunctional properties make it particularly effective in improving soil physicochemical characteristics, promoting plant growth, and alleviating the adverse effects of environmental stressors [39,40,41,42]. Most previous studies have focused on the source-based classification of HA, emphasizing its origin and general agronomic benefits. However, there is a notable gap in comprehensive reviews that examine the structure–function relationship of HA, specifically, how variations or modifications in its molecular structure can influence its biological activity and broaden its potential applications. In this review, we provide an in-depth analysis of the structural features of HA and their relevance to its functional roles in plants. We explore its mechanisms in mediating stress tolerance and discuss innovative approaches for structural modification aimed at enhancing its efficacy. By addressing these aspects, this study offers valuable insights into the targeted use of HA to improve plant resilience against diverse environmental stress conditions.

This review distinguishes itself by focusing on the structure–function relationship of HA, providing an in-depth analysis of how specific structural features influence their biological activity and enhance enzymatic responses that mitigate abiotic stress in plants. While earlier reviews have often treated HA as a generic soil amendment or fertilizer additive, our work bridges the gap between structural chemistry and functional outcomes in plant systems. We highlight recent advancements in HA surface modification techniques and their implications for plant stress physiology. By integrating both established knowledge and emerging research, this review offers a mechanistic framework for understanding HA–plant interactions. Such insights are crucial for the rational design of HA-based biostimulants and for promoting sustainable agricultural practices. Ultimately, this review aims to support researchers and practitioners in harnessing the full potential of HA for more targeted and effective crop management strategies under environmental stress conditions.

## 2. Humic Acid Molecular Structure and Functional Groups

Humic acid is a natural organic fertilizer characterized by a complex molecular structure that includes functional groups such as carboxyl (–COOH), alcoholic (–OH), carbonyl (C=O), and phenolic (Ar-OH) moieties [43]. These groups play a key role in enhancing nutrient transport and availability, as well as improving the physicochemical properties of soil. Organic acids released through root exudates or microbial activity can interact with HAs, altering their molecular weight and conformation. These interactions affect the HAs’ reactivity and solubility, thereby influencing nutrient dynamics and overall soil chemistry [16]. Beyond soil enhancement, HA also stimulates various biochemical processes within plants. It has been shown to boost photosynthesis and respiration, enhance the production of hormones and proteins, and ultimately promote plant growth and productivity [44,45]. According to Dinçsoy and Sönmez [46], HA generally exerts a positive effect on plant physiology by encouraging root development and improving nutrient uptake. Collectively, this evidence underscores the crucial role of HA in improving soil fertility and positively influencing plant physiological functions.

It is obvious that HA is often linked to improvements in soil physicochemical properties [25], although there are varying perspectives concerning its effects on crop physiology. For example, Rose et al. [26] revealed that the source of HA affected the growth of shoots and roots, whereas the application rate of HA was observed to affect only shoot growth, indicating that concentration primarily impacts aerial development, while root growth remains unaffected. On the other hand, some studies have also reported no significant effects on crop growth and soil physiochemical properties after HA application [22,23,24]. de Melo et al. [10] demonstrated that carboxyl (–COOH) and phenolic (–OH) groups in HA are mostly responsible for soil functions such as nutrient retention and microbial activity. The effectiveness of HA in soil and plant growth depends on its source, chemical composition, molecular structures, and application rate [19]. However, the use of HA can lead to inconsistent yield outcomes, most likely due to variations in biological sources that affect its chemical composition, structural integrity, and functional properties. These differences influence nutrient availability, soil interactions, and plant growth responses, resulting in unpredictable effects on productivity [12].

## 3. Structure–Function Relationships of Humic Acid

The functions of HA are closely linked to its source-dependent structural characteristics. Among the most common and functionally important groups present in HA are hydroxyl and carboxyl groups [19]. The spatial arrangement and dissociation of these groups give rise to hydrophilic and hydrophobic domains, both of which are crucial to HA’s beneficial effects [47]. The hydrophilic regions, rich in polar functional groups, facilitate metal chelation and complex formation, while the hydrophobic regions contribute to water repellency and structural stability in soil [11]. Upon dissociation, the carboxyl and hydroxyl groups confer negative charges to HA molecules, enabling them to electrostatically bind with positively charged metal ions, thus forming stable organo-metal complexes. The hydrophilic domains also promote micelle formation, enhancing the soil’s water-holding capacity. In contrast, the hydrophobic domains help stabilize clay aggregates by repelling water molecules and limiting excessive water infiltration. According to van Tol de Castro et al. [48], the functional groups in HA contribute to increased nitrogen uptake and accumulation of soluble sugars, ultimately enhancing rice yield. Similarly, García et al. [49] reported that these groups stimulate root development in rice seedlings by improving nutrient availability and interacting with root cell membranes (mechanisms that help plants cope with stress conditions). These findings are supported by Piccolo [50], who demonstrated that HS enhance nutrient mobility and uptake through the activation of root membrane functions, and by Garcia-Mina et al. [51], who revealed HS-induced modulation of root nutrient transport systems. Additionally, Hayes and Swift [52] emphasized that the conformational flexibility of HS is key to regulating mineral interactions and nutrient availability. Collectively, these studies underscore the essential role of HS in optimizing nutrient acquisition and improving plant resilience under abiotic stress conditions.

Chemical modification of HA has emerged as a valuable strategy to enhance its functional properties and broaden its agricultural and environmental applications [53]. Among these approaches, nitrogen functionalization is particularly promising for increasing HA bioactivity and nutrient interaction capabilities [54]. Introducing specific catalysts during the oxidation and modification of HAs can significantly improve the yield and quality, as well as the degree of functionalization [55,56]. One such advancement involves the development of nitrogen-functionalized HAs (NHAs), which incorporate reactive nitrogen groups, such as nitro (–NO_2_), nitroso (–NO), amide (–CONH_2_), and amine (–NH_2_), into the HA molecular structure [55]. This functionalization is typically achieved through oxidative processes using agents such as nitric acid (HNO_3_), hydrogen peroxide (H_2_O_2_), and, more recently, ozone [57,58,59,60,61,62]. A range of methods, including physical, chemical, and physicochemical techniques, can be employed to synthesize NHAs effectively [63].

One of the most promising methods for synthesizing NHAs involves the physicochemical transformation of organic waste through a solid-phase nitro-humification process [54]. This approach combines nitrogen doping with ozone oxidation, enabling the incorporation of slow-release nitrogen forms, such as amide-N and organically bound nitrogen into the HA structure, while activating up to 70% of alkali-soluble HA [55]. This method not only increases the concentration of functional groups but also improves the critical physicochemical properties of HA, such as cation exchange capacity and hydrophilicity, which contribute to maintaining soil osmotic balance [64]. Moreover, it delivers a well-balanced profile of nutrients and rare earth elements while reducing the heavy metal content in the final product. The process also yields a material with a high mesopore density and a negatively charged surface, enhancing sorption capabilities. Compared to traditional alkaline extraction methods, this technique is more cost-effective and significantly improves the overall functional efficiency of HA [55].

## 4. Humic Acid-Mediated Stress Alleviation in Plants

**Figure 3:**
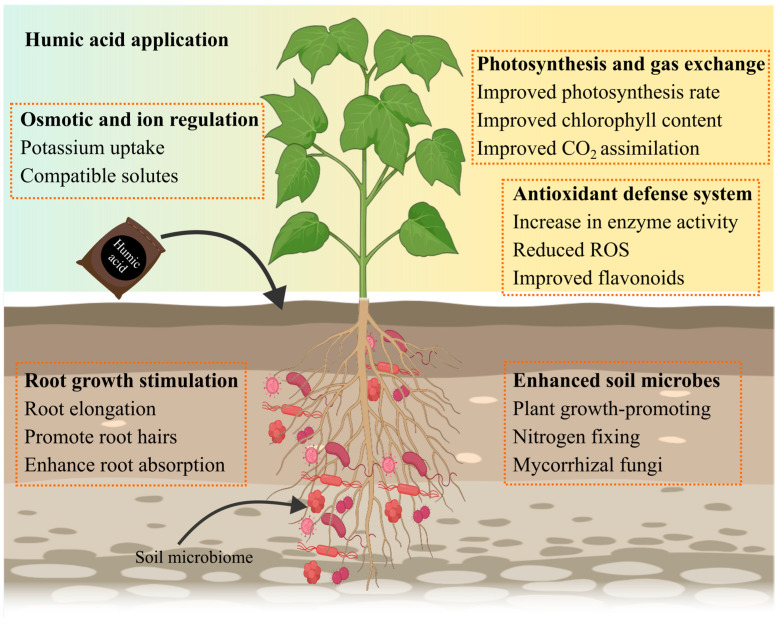
Beneficial effects of HA on soil physiochemical characteristics (osmotic and ion regulation, cation exchange capacity, and soil microbial population), growth morphology, physiology (photosynthesis, chlorophyll content, and CO_2_ assimilation), and biochemistry of plants.

Plants are continually exposed to various environmental stressors in their natural habitats, including drought, salinity, extreme temperatures, and heavy metal contamination [65]. These abiotic stresses can significantly hinder plant growth and productivity. A common consequence of such stressors is the induction of oxidative stress, characterized by excessive production of ROS, such as superoxide (O_2_^−^), hydrogen peroxide (H_2_O_2_), and singlet oxygen (^1^O_2_). Elevated ROS levels disrupt cellular homeostasis, leading to enzyme inhibition, chlorophyll degradation, lipid peroxidation, and overall biomolecular damage [66]. Recent studies highlight the pivotal role of HS, particularly HA, as regulators of both primary and secondary plant metabolism. HA has shown considerable promise in mitigating the adverse effects of abiotic stress and enhancing plant resilience (Figure 3). For instance, Kaya et al. [67] demonstrated that foliar application of HA in pepper plants (*Capsicum annuum* L.) improved salt tolerance under 100 mM NaCl conditions. The treatment increased proline and chlorophyll content, along with the activities of key antioxidant enzymes such as superoxide dismutase (SOD), catalase (CAT), and peroxidase (POD). In beans, foliar HA application had differential effects on antioxidant enzyme activity, decreasing CAT and ascorbate peroxidase (APX), while enhancing SOD and glutathione reductase (GR) activity [68]. Amerian et al. [42] further explored a combined strategy involving HA application and grafting onto salt-tolerant rootstocks in cucumber. This integrative approach led to improvements in plant growth, yield, photosynthetic performance, antioxidant enzyme activity, and the accumulation of secondary metabolites under salt stress conditions. Notably, it also reduced sodium (Na^+^) and chloride (Cl^−^) accumulation in shoots, while enhancing the uptake of essential nutrients like potassium (K^+^) and calcium (Ca^2+^). At higher salinity levels, this combined treatment promoted the biosynthesis of secondary metabolites, such as flavonoids and phenolic acids, alongside improved relative water content (RWC) and reduced electrolyte leakage. Salt stress typically triggers Na^+^ extrusion from the cytosol via efflux mechanisms, including Na^+^/H^+^ antiporters and the salt overly sensitive signaling pathway, which help maintain ionic balance across the plasma membrane [69]. Once Na^+^ enters root cells and is transported to aerial tissues, vacuolar sequestration becomes essential to prevent cytosolic toxicity [70,71]. This sequestration is aided by Na^+^/H^+^ antiporters (NHX), which mediate ion exchange across vacuolar membranes. Members of the high-affinity potassium transporter (*HKT*) family remove Na^+^ from the xylem, reducing its movement or accumulation in the plant’s aerial parts [72]. Furthermore, it was shown that the activity of *HKT1* transporters was boosted, allowing Arabidopsis to endure the negative effects of salt [73]. Importantly, HA treatment has been found to activate many components of the ion homeostasis machinery, even in the absence of high external Na^+^ concentrations, suggesting its role in pre-conditioning plants for enhanced stress resilience.

In a similar study, Alsudays et al. [74] explored the role of HA, FA, and the recommended dose of phosphorus fertilizer (RDP) in enhancing barley (*Hordeum vulgare* L.) seedlings under saline conditions. The combination of HA + 100% RDP demonstrated the greatest improvements in plant growth metrics such as grain yield, straw yield, spike weight, and nutrient uptake in both seasons. Grain yield increased by up to 64.69% over the control and by 22.30% compared to RDP alone. These results highlight the synergistic effects of HA and FA in mitigating salinity stress, improving nutrient availability, and enhancing barley resilience and productivity, making these organic amendments valuable tools for sustainable agriculture in saline environments. Malik et al. [75] also investigated the combined effects of rice-straw biochar (1%) and HA (0.15%) on maize growth under salinity stress (6 and 12 dS/m NaCl). Salinity significantly decreased growth, biomass, chlorophyll content, RWC, and antioxidant enzyme activity, while increasing Na^+^ intake, oxidative stress, and lipid peroxidation. The combined application of biochar and HA significantly promoted both shoot and root growth, enhanced root architecture, and increased RWC by 14.8%. It also led to a 12.2% reduction in Na^+^ accumulation in leaf tissues. By strengthening the plant’s antioxidant defense system, this treatment lowered oxidative damage, as evidenced by reductions of up to 41.2% in malondialdehyde (MDA) and hydrogen peroxide (H_2_O_2_) levels. Moreover, an improved K^+^/Na^+^ ratio contributed to maintaining ionic homeostasis. Collectively, these physiological and biochemical enhancements underscore the effectiveness of the biochar–HA combination in mitigating salt stress and offer a sustainable long-term strategy for enhancing crop resilience in saline environments.

Focusing on the impact of HA under drought stress, Abu-Ria et al. [76] investigated its effects on *Sorghum bicolor* (L.) Moench (sorghum) and *Zea mays* L. (maize). The study revealed that drought stress significantly reduced plant growth, photosynthetic pigment levels, and RWC, while increasing oxidative stress in both species, with maize exhibiting greater sensitivity. However, HA seed-priming under drought conditions effectively stimulated growth, enhanced photosynthetic efficiency, and improved key metabolic indicators. In maize, HA application notably improved water retention and nutrient uptake, reduced oxidative damage, and boosted antioxidant enzyme activity, thereby mitigating the adverse effects of drought stress. Bijanzadeh et al. [77] investigated the combined application of HA and salicylic acid (SA) to alleviate drought stress in two maize hybrids (SC 260 and SC 705) grown under hydroponic conditions. Drought stress led to increased electrolyte leakage, reduced levels of photosynthetic pigments and RWC, and impaired root and shoot development. Among the two hybrids, SC 705 exhibited greater drought tolerance. The combined treatment of HA and SA enhanced drought resistance by improving membrane stability, increasing chlorophyll content and RWC, enlarging root xylem dimensions, and boosting K accumulation effects, which were particularly pronounced in the more tolerant SC 705 hybrid.

Similar to drought, heavy metals stress is more frequent in agricultural soils. In a similar situation, Wang et al. [78] investigated the effects of HA on mitigating cadmium (Cd) stress on sunflowers. They demonstrated that the Cd stress reduces growth and photosynthetic pigments, and increases oxidative stress in plants. Moreover, HA reduces the ROS content (O_2_^−^, H_2_O_2,_ and OH^−^), which has a positive effect on sunflowers and alleviates Cd-induced stress. Similarly, Yildirim et al. [79] used HA and FA to evaluate the Cd toxicity in *Lepidium sativum* L. (garden cress). The combined HA + FA applications reduced the adverse effects of the Cd contamination, and the plants showed better growth and physiological and biochemical responses, including decreased oxidative stress. Wen et al. [80] used a combination of pyrite and HA to assess the toxicity of arsenate in *Lactuca sativa* L. (lettuce) (Table 1).

The results showed that the combined application of pyrite and HA immobilized more arsenic (As) by forming chemical connections such as As-S and Fe-As-O, decreasing As transport to plants. Furthermore, a metabolomics investigation found that the V-PF group facilitated glycolysis by enhancing glyoxylic acid and dicarboxylic acid metabolism, resulting in decreased carbohydrate accumulation. Phosphocreatine metabolism was also enhanced, reducing As-induced oxidative stress in lettuce. Duan et al. [96] examined the HA’s regulatory mechanisms on lead (Pb) stress in *Camellia sinensis* L. via different techniques such as scanning transmission X-ray microscopy, transmission electron microscopy, and isobaric tags for relative and absolute quantitation differential proteomics. The results revealed that HA significantly reduced Pb stress and enhanced the levels of pectic acid and pectin in the cell wall, while arabinose and galactose decreased, which was beneficial to enhancing Pb absorption. Furthermore, isobaric tags for relative and absolute quantitation (iTRAQ) analysis demonstrated that HA increased the antioxidant system activity, accelerated cell wall formation, and stabilized the metabolism of protein and sulfur-containing substances at the molecular level. Ran et al. [97] investigated the effects of HA and FA from different sources on the influence of mercury (Hg) methylation in the soil and its accumulation in rice. HA enhances soil Hg methylation and reduces Hg mobility in the plant. However, it was also revealed that HA increases methylmercury accumulation in rice grains, raising concerns about their feasibility as organic modifiers in Hg-contaminated soils. In addition to increasing the soil’s water content and P, K, iron (Fe), and magnesium (Mg) availability, the use of HA increased the proportion of macro-aggregates. In maize, the levels of osmotically active solutes (soluble sugars, betaine, and proline), the activity of Rubisco and ATP synthase, and the indole acetic acid (IAA) content all increased. Furthermore, genes related to metabolic activities, including photosynthesis, carbon fixation, hormones, and osmotic metabolisms, were expressed more often in maize leaves [87].

Heavy metal-induced oxidative stress, much like the stress caused by salinity, drought, and extreme temperatures, poses a substantial challenge to plant growth and productivity. HA has proven to be highly effective in mitigating such stresses, primarily due to the existence of carboxyl and phenolic groups. These groups act as effective natural antioxidants, actively sifting ROS that accumulate under heavy metal stress. By neutralizing ROS, HA reduces oxidative damage to dynamic cellular components such as lipids, proteins, and nucleic acids, thereby maintaining cellular integrity and functionality. In addition to its ROS-scavenging ability, HA stabilizes plant cell membranes, reducing electrolyte leakage and preserving cellular homeostasis under stressful conditions. Its action helps enhance the activity of endogenous antioxidant enzymes such as SOD, CAT, and POD, further stimulating the plant’s defense system [98,99]. Moreover, HA improves nutrient availability and uptake, which is often reduced under heavy metal stress, ensuring that plants have access to essential elements for growth and repair. These combined effects not only alleviate oxidative stress but also enhances overall plant growth and yield [77].

## 5. Mechanism of Humic Acid-Mediated Stress Alleviation

**Figure 4:**
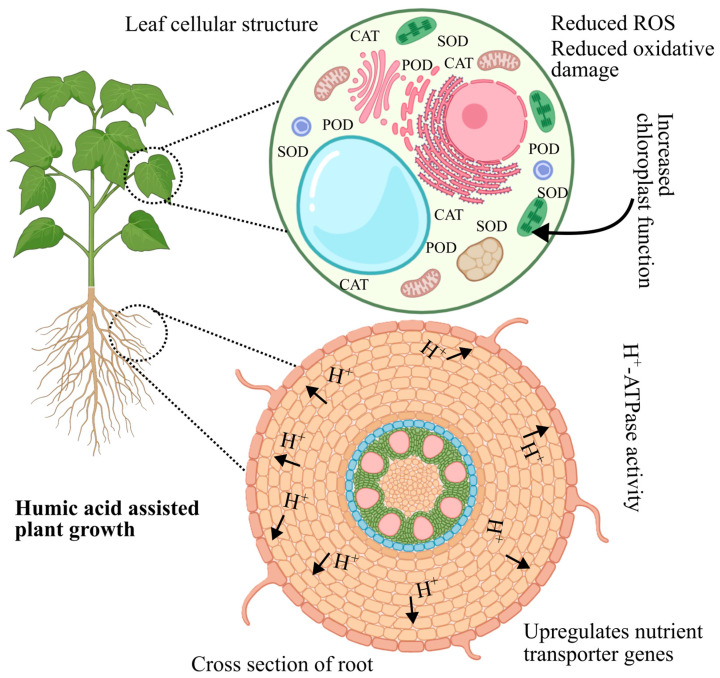
HA-mediated ROS scavenging: a mechanistic overview of oxidative stress mitigation through extrusion of ROS from plant cells and stress alleviation.

Plants’ HA-mediated abiotic stress relief mechanism is complex, including multiple physiological and biochemical processes. HA is a natural biostimulant that mimics plant hormones including auxins, gibberellins, and cytokinins, promoting root elongation, branching, and the production of lateral roots and root hairs [39]. This expanded root system enhances water and nutrient uptake, improving plant resilience under stress. HA also facilitates the absorption of essential ions like K, maintaining osmotic balance and cellular turgor. Additionally, it promotes the production of compatible solutes like proline and carbohydrates, which stabilize proteins and membranes during stress conditions [15,65,100]. HA increases photosynthetic efficiency by enhancing chlorophyll synthesis and reducing pigment degradation, ensuring optimal CO_2_ assimilation and water use efficiency (WUE). It enhances the antioxidant defense system by raising the activity of enzymes such as SOD, POD, and CAT, as well as non-enzymatic antioxidants like phenolics and flavonoids, which minimize oxidative damage produced by ROS (Figure 4). Furthermore, HA improves soil quality by fostering beneficial interactions with microbes, including nitrogen-fixing bacteria, phosphate-solubilizing microorganisms, and rhizobacteria that promote plant growth. These microorganisms increase the availability of important nutrients like nitrogen and P, which help plants cope with abiotic stress [101,102].

## 6. Humic Acid Modification for Stress Alleviation

**Figure 5:**
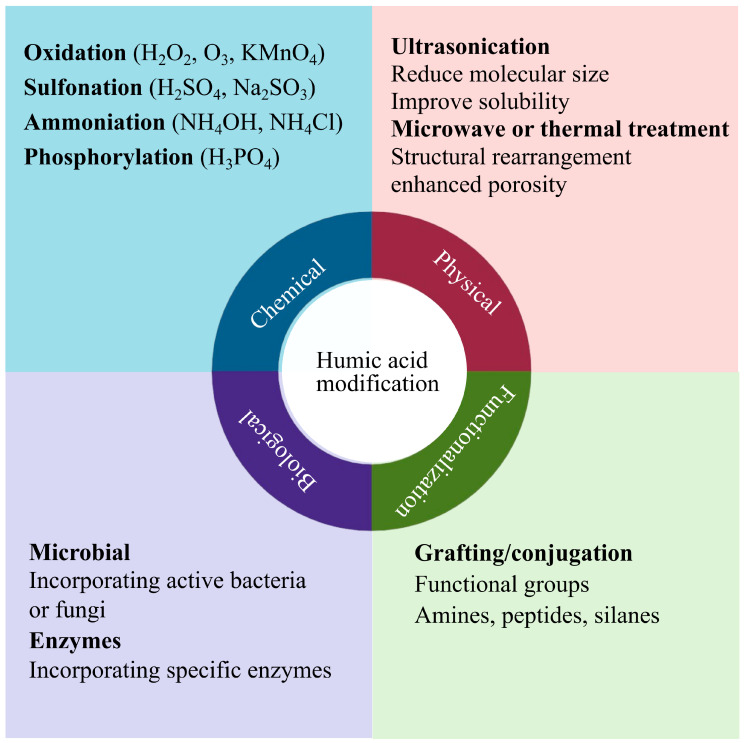
Categories and mechanisms of humic acid modification to enhance plant stress alleviation.

The functionality of HA can be significantly enhanced through a range of chemical, physical, biological, and functionalization strategies (Figure 5), rather than relying solely on surface-level modifications. Due to the complex, three-dimensional, and often fractal architecture of HA macromolecules (with reactive groups variably embedded within or exposed on the surface) scientific consensus now favors describing improvements in overall functionality or the abundance and nature of reactive groups, rather than referring simply to “surface modification”. Enhancing specific functional groups, such as carboxyl (–COOH/–COO^−^), hydroxyl (-OH), and phenolic moieties, can greatly improve HA solubility, nutrient chelation capacity, and interaction with plant root systems (Table 2) [21]. For instance, an increase in carboxyl group content enhances nitrogen uptake and nutrient availability by promoting stronger metal ion chelation and more efficient microbial interactions [103,104]. To optimize HA for agricultural applications, various modification techniques can be employed. These include chemical modifications (e.g., oxidation, sulfonation), physical treatments (e.g., ultrasonication, thermal processing), biological methods (e.g., enzymatic or microbial incorporation), and functionalization strategies (e.g., grafting with amines, peptides, or silanes), as illustrated in Figure 5. These approaches aim to adjust molecular size, increase porosity, enhance solubility, and introduce or amplify beneficial functional groups, ultimately improving HA’s stability and its effectiveness in plant–soil systems. For example, chemical oxidation using agents like potassium permanganate (KMnO_4_) or ozone (O_3_) can elevate the content of oxygen-containing functional groups [61,62], while ultrasonication can reduce molecular size, thereby increasing HA solubility and bioavailability.

Among recent advances, nano-humic acid (nano-HA) formulations have gained attention for their enhanced delivery efficiency and bioactivity. However, the environmental and human health implications of nano-HA remain insufficiently understood. Due to their small size and high reactivity, nanoparticles can persist in the environment, accumulate in soil and water systems, and potentially disrupt microbial communities or enter the food chain. Emerging studies suggest that chronic exposure to engineered nanomaterials may pose risks to non-target organisms and raise biosafety concerns [105,106]. Thus, while nano-HA technologies hold significant promise, it is critical to assess their long-term ecological impacts, bioaccumulation behavior, and safe dosage thresholds. Integrating nanotechnology with sustainability principles is essential to avoid unintended consequences while maximizing agricultural benefits. Although biochar is often considered low in extractable HA, this is not only due to its recalcitrant structure but also to the loss of oxygen-containing functional groups such as –COOH and –OH during the thermal preparation process; however, this generalization does not apply to all types of biochar. Feedstock type and pyrolysis conditions strongly influence the presence and composition of HS [107,108,109]. For example, Laskosky et al. [20] compared HA from humalite, biochar, and peat in barley and found that humalite and peat, with higher initial nitrogen and P, improved growth and yield more than biochar. While existing studies demonstrate the potential of modified HAs in improving plant growth and stress tolerance, comprehensive comparisons across HA sources and modification strategies remain limited.

Humic acid can be biologically modified using specific microbes or enzymes to introduce or increase functional groups (e.g., carboxyl, hydroxyl), improve molecular complexity, and promote a more reactive structure, thereby enhancing its overall functionality and bioactivity [110]. Microorganisms such as bacteria and fungi can metabolize and transform HA, altering its molecular composition and increasing the abundance of functional groups like carboxyl, hydroxyl, and phenolic moieties. This microbial action can improve HA solubility, nutrient-binding capacity, and interaction with plant roots [111]. Enzymes such as lignin peroxidases and laccases (often produced by these microbes) facilitate the oxidative breakdown and restructuring of HA molecules, introducing new reactive sites and lowering molecular weight, which enhances HA’s mobility and bioavailability in soil [112]. These biotransformations not only improve HA’s efficacy as a plant growth enhancer and soil conditioner but also align with environmentally friendly and sustainable agricultural practices. Future studies should focus on evaluating the structure–function relationships of HAs from diverse origins and treatments under controlled and field conditions to better understand their physiological and biochemical impacts.

Recent advancements have introduced nano-catalyst-assisted mechanochemical activation as a sustainable and highly efficient approach to enhance the reactivity and solubility of HA. Unlike traditional methods that rely on harsh chemical oxidants, this technique employs nano-catalysts to intensify the activation process in a more environmentally friendly manner. For example, nitrogen-rich humic fertilizers can be synthesized using extrusion-based mechanochemical techniques, which increase the abundance and accessibility of reactive functional groups. This not only promotes greater nitrogen uptake by plants but also reduces nitrogen losses through leaching and enhances soil water retention [113]. Furthermore, enriching HA with essential metals such as calcium and magnesium improves the overall nutritional quality of the fertilizer and enhances plant health and resilience under stress conditions [113,114]. Moreover, embedding bioactive compounds such as polyphenols or flavonoids enhances the antioxidant capacity of HA, enabling more effective scavenging of ROS and mitigating oxidative damage in plants. Finally, the chemical incorporation of metal ions, such as zinc (Zn) and iron (Fe), or sodium (Na) enhances HA’s metal-chelating properties by occupying specific functional groups and stabilizing its structure, which improves its capacity to bind and immobilize toxic heavy metals. Additionally, Zn and Fe are essential micronutrients that, when complexed with HA, are more bioavailable to plants, promoting root development and nutrient uptake. Na can improve HA solubility and cation exchange capacity, facilitating nutrient transport in the rhizosphere. Together, these effects help alleviate heavy metal toxicity and support plant growth under adverse environmental conditions [115,116].

**Table 2:** Functional groups of humic acids and their associated plant benefits.

**Functional Group**	**Associated Chemical or Biological Modifications**	**Plant Benefit**	**Ref**
–COOH (Carboxyl Acid)	Promotes interaction with plant growth-promoting rhizobacteria (PGPR), and arbuscular mycorrhizal fungi (AMF)	Enhances antioxidant production, improves growth and abiotic stress tolerance	[117]
–OH (Hydroxyl Group)	Antioxidant-modulated pathways	Enhances soil moisture retention and scavenging of ROS	[118]
Aromatic Rings	Modified by sulfonation or phenolic enrichment	Increases hydrophilicity, contributes to structural stability and stress resilience	[119,120]

## 7. Conclusions

Humic acids is a complex, multifunctional macromolecule that plays a pivotal role in improving soil physicochemical properties, promoting plant growth, and mitigating abiotic stress. This review provides a comprehensive overview of HA’s structural features and its physiological and biochemical mechanisms supporting plant health and resilience. Emerging surface modification strategies targeting key functional groups involved in ROS scavenging, nutrient chelation, and hormonal signaling offer promising routes to enhance HA’s bioefficacy under stress conditions. Such advances have the potential to contribute significantly to sustainable and precision agriculture. While modified forms of HA, including nano-scale variants, show promise for enhanced functionality, their environmental and biosafety implications require careful consideration. Further systematic studies are essential to evaluate their long-term effects on soil ecosystems, non-target organisms, and human health before broad agricultural application.

Future research should focus on elucidating the structure–function relationships of modified HA at molecular and nano-scale levels to enable more targeted applications. Additionally, investigating synergistic interactions between HA and other biostimulants or soil amendments could optimize its benefits for plant and soil health.

## Figures and Tables

**Table 1:** Effects of humic acid on crop performance under various stress conditions.

**Stress Type**	**Crop**	**Environment**	**HA Form & Dose**	**Application Details (Frequency, Duration, Volume/Area)**	**Key Findings**	**Ref**
Salinity	*Zea mays* L. (maize)	Greenhouse	HA, 50 mg L^−1^	Single application; duration not reported	Increased seed germination and seedling growth under salinity	[81]
	*Chenopodium quinoa* L. (quinoa)	Field	HA, 1% (*v*/*v*)	Weekly application; 60 days; approx. 1 L/m^2^	Increased plant height, fresh weight, and dry matter	[82]
	*Oryza sativa* L. (rice)	Greenhouse	HA, 100 mg L^−1^	Applied at transplant; duration 30 days	Enhanced antioxidant enzyme activity and root growth	[83]
	*Triticum aestivum* L. (wheat)	Greenhouse	HA, 200 mg kg^−1^ soil	Single soil application; 45 days	Increased yield and productivity	[84]
	*Carica papaya* L. (papaya)	Greenhouse	HA, 3.5 mL L^−1^	Biweekly foliar spray; 8 weeks	Alleviated salt stress, promoted growth, and improved photosynthesis	[85]
	*Carica papaya* L. (papaya)	Greenhouse	HS, 20 g kg^−1^	Soil mixed before planting; 60 days	Increased photosynthesis, CO_2_ assimilation., WUE, and chlorophyll in saline conditions	[86]
Drought	*Zea mays* L. (maize)	Greenhouse	HA, 45 kg ha^−1^	Soil application at sowing; 90 days	Improved nutrient availability, WUE, Rubisco activity, sugars, and osmolyte content	[87]
	*Glycine max* L. (soybean)	Greenhouse	HA, 5 mg dm^−3^	Applied at sowing; 45 days	Increased antioxidant enzyme activity, biomass, and root length	[88]
	*Capsicum annuum* L. (pepper)	Field	HA, 4.5 L ha^−1^	Weekly irrigation with HA; 75 days	Enhanced shoot biomass and growth under drought	[89]
Extreme Temp.	*Solanum lycopersicum* L. (tomato)	Laboratory	HA, 500 mg L^−1^	Foliar spray before heat exposure	Improved growth, fluorescence, antioxidant activity, and heat-responsive gene expression	[90]
	*Arabidopsis thaliana*	Laboratory	Commercial HA, 860 mg L^−1^	Single spray before heat stress	Enhanced heat stress tolerance via HSP gene expression	[91]
	*Coriandrum sativum* L. (coriander)	Greenhouse	HA, 50 mg L^−1^	Applied during irrigation; 30 days	Promoted growth, enhanced antioxidants, and secondary metabolites	[92]
Heavy Metals	*Triticum aestivum* L. (wheat)	Field	HA, 40 mg kg^−1^	Soil amendment before planting; 60 days	Increased biomass, reduced oxidative stress under Cd stress	[93]
	*Fragaria × ananassa Duch.* (strawberry)	Greenhouse	HA, 5 mM	Foliar application; 45 days	Reduced Cd toxicity via improved membrane stability and increased proline	[94]
	*Brassica napus* L. (rapeseed)	Greenhouse	HA, 2000 mg kg^−1^	Soil amendment; 60 days	Increased growth, reduced metal accumulation and oxidative stress	[95]
	*Lepidium sativum* L. (garden cress)	Greenhouse	HS, 7000 mg L^−1^	Hydroponic treatment; 20 days	Increased biomass and root diameter; reduced Cd uptake by up to 95%	[79]

Note: HA = humic acid; HS = humic substances; WUE = water use efficiency. Application details (frequency, duration, volume/area) are reported when available; some studies did not specify all these parameters. Humic acid formulations may vary (e.g., dissolved in KOH, colloidal suspension, or gel form), which could affect comparability across studies.
